# Hypertonic saline induces inflammation in human macrophages through the NLRP1 inflammasome

**DOI:** 10.1038/s41435-023-00218-7

**Published:** 2023-08-12

**Authors:** Francesca Sposito, Sarah Northey, Amandine Charras, Paul S. McNamara, Christian M. Hedrich

**Affiliations:** 1 Department of Women’s & Children’s Health, Institute of Life Course and Medical Sciences, University of Liverpool, Liverpool, UK; 2 Department of Respiratory Medicine, Alder Hey Children’s NHS Foundation Trust Hospital, Liverpool, UK; 3 Department of Paediatric Rheumatology, Alder Hey Children’s NHS Foundation Trust Hospital, Liverpool, UK

**Keywords:** Inflammasome, Monocytes and macrophages

## Abstract

Nebulized hypertonic saline (3–7%) is commonly used to increase mucociliary clearance in patients with chronic airway disease and/or virus infections. However, altered salt concentrations may contribute to inflammatory responses. The aim of this study was to investigate whether 500 mM NaCl (3%) triggers inflammation in human macrophages and identify the molecular mechanisms involved. NaCl-induced pyroptosis, IL-1β, IL-18 and ASC speck release were measured in primary human monocyte-derived macrophages. Treatment with the recombinant IL-1 receptor antagonist anakinra or the NLRP3 inhibitor MCC950 did not affect NaCl-mediated inflammasome assembly. Knock-down of *NLRP1* expression, but not of *NLRP3* and *NLRC4*, reduced NaCl-induced pyroptosis, pro-inflammatory cytokine and ASC speck release from human THP-1-derived macrophages. Data from this study suggest that 3% NaCl-induced inflammatory responses in human macrophages depend on NLRP1 and inflammasome assembly. Targeting inflammation in addition to inhalation with hypertonic saline may benefit patients with inflammatory airway disease.

## Introduction

The luminal surface of airway epithelia is covered by mucus. Its function is to trap inhaled microbes and particles and allow their clearance. Inhalation of nebulized hypertonic saline (usually 3–7% NaCl) is used therapeutically to enhance airway clearance in respiratory inflammatory conditions such as cystic fibrosis or bronchiolitis [1]. In vitro, hypertonic saline improves mucus elasticity and viscosity and in vivo, mucus hydration, thereby facilitating its expulsion through coughing [2]. The effectiveness and safety of this treatment, however, remain unclear [2–6]. Notably, although short-term clinical efficacy has been demonstrated, increased airway salt concentrations may have damaging effects through osmotic stress and resulting inflammation [7].

Bronchial epithelia and immune cells closely interact in the bronchoalveolar environment. Both immune and epithelial cells express inflammasomes and associated genes [8]. Inflammasomes are cytoplasmic multi-protein complexes that assemble upon activation. As soon as a cytoplasmic receptor senses “danger signals”, inflammasomes oligomerize. Inflammasomes include their name-giving cytoplasmic sensor (e.g., NLRP1, NLRP3 (NLR Family Pyrin Domain Containing 1 & 3), NLRC4 (NLR Family CARD Domain Containing 4)), (usually) the adaptor molecule apoptosis-associated speck-like protein containing a CARD (ASC), and (pro-)caspase-1 [8]. Once activated, caspase-1 cleaves immature cytoplasmic cytokine precursors pro-IL-1β and pro-IL-18, and gasdermin D into their active forms, resulting in the release of highly potent IL-1β and IL-18, and inflammatory cell death, so-called pyroptosis [8, 9]. Abnormally high intracellular ionic concentrations and altered extracellular osmolarity can activate NLRP1, NLRP3 and NLRC4 inflammasomes [10–14]. Of note, NLRP1 has been linked to inflammation in a mouse model of dextran sulfate sodium salt-induced colitis; increased cytoplasmic sodium concentrations activate NLRP3; and both NLRP3 and NLRC4 are hyperosmotic stress sensors [10, 13, 14]. Thus, hypertonic saline may induce inflammasome assembly and pro-inflammatory cytokine release, thereby worsening/aggravating airway inflammation and bronchopulmonary disease.

The aim of this study was to investigate/determine whether 3% hypertonic saline triggers inflammation in human macrophages, and to identify the molecular mechanisms involved.

## Materials and methods

### Cell culture

Primary human monocytes were isolated from peripheral blood of eight healthy donors (Table 1) following a three-step process, including HetaSep (StemCell, UK, cat. 07096), Histopaque-1077 (Sigma-Aldrich, cat. 10771), and the Human Monocytes Isolation Kit (StemCell, UK, cat. 19359). Monocytes were cultured in vitro at 37 °C with 5% CO_2_ in complete RPMI medium (Gibco, cat. 61870010, supplemented with 10% FBS and 100 U/mL Penicillin-Streptomycin) at 1 × 10^6^ cells/mL in the presence of 25 ng/ml M-CSF (Peprotech, cat. 300-25). Cells were cultured for 6 days to allow differentiation into macrophages. Experiments were performed on day 7. This study was approved by the University of Liverpool Ethical Committee and informed consent for participation was obtained from all donors.

**Table 1:** Donors’ demographics.

Donor	Sex	Age
D1	F	30
D2	F	35
D3	F	34
D4	M	25
D5	M	36
D6	F	50
D7	M	43
D8	M	46

Human THP-1 monocytes (leukemia monocytic cell line, ATCC) were cultured at 37 °C with 5% CO_2_ in complete RPMI at a concentration of 1 × 10^5^ cells/mL. The day before stimulations, cells were seeded in 96-wells plates at a concentration of 1 × 10^6^ cells/mL in RPMI complete medium with the addition of 2.5 ng/mL phorbol 12-myristate 13-acetate (PMA) to allow differentiation into macrophage-like cells. The absence of mycoplasma infection in the cell cultures was confirmed monthly by targeted PCR.

### Knock-down experiments

The day after seeding, THP-1 cells were transfected using the Lipofectamine RNAiMAX Transfection Reagent (Invitrogen, cat. 13778030) and 20 pmol of small interfering (si) RNAs targeting NLRP1, NLRP3 or NLRC4. Scrambled siRNA was included as a control (OriGene, Table 2). Stimulations were performed 24 h after transfection. Seven independent experiments were performed, with two technical replicates/condition.

**Table 2:** Small interfering (si)RNAs.

Target gene	Name	Origene cat. number
*NLRP1*	NALP1 (NLRP1) Human siRNA Oligo Duplex (Locus ID 22861)	SR323441
*NLRP3*	NLRP3 Human siRNA Oligo Duplex (Locus ID 114548)	SR325572
*NLRC4*	CARD12 (NLRC4) Human siRNA Oligo Duplex (Locus ID 58484)	SR324786
Scrambled siRNA (negative control)	Trilencer-27 Universal scrambled negative control siRNA duplex	SR30004

### Stimulations

On the day of the experiment, phenol red-free complete RPMI (Gibco, cat. 32404014), with or without 100 ng/mL *Escherichia coli* lipopolysaccharide (LPS, Sigma-Aldrich, Dorset, UK) and/or 500 mM NaCl (Sigma) was added to the cells as indicated. Notably, 500 mM NaCl resembles 3% saline solution frequently used for inhalation treatment [15]. Cell supernatants were collected 8 hours after stimulation.

### Inhibitors

The recombinant IL-1 receptor antagonist anakinra (SOBI, Stockholm, Sweden) was added to complete RPMI without phenol red to a final concentration of 10 μg/mL. The small molecular NLRP3 inhibitor MCC950 (Sigma-Aldrich, Dorset, UK) was added to a final concentration of 10 μM. Either anakinra or MCC950 were added to the cell cultures 30 min prior stimulation with LPS and/or NaCl, and again together with the stimulation agent/agents.

### Inflammatory cell death assay

Cell culture supernatants (50 μL) were used to perform the LDH assay using the Cytotoxicity Detection Kit PLUS (Roche Diagnostics Deutschland GmbH, cat. 11644793001). Absorbances (Abs) were measured using a POLARstar Omega spectrophotometer (BMG LABTECH, Ortenberg, DE). The percentage of cell death was evaluated using a positive control were all cells were lysed using the kit’s lysis solution using the following formula:

$${{{\mathrm{\% }}}}\,cell\,death = \frac{{Abs_{test\,sample} - Abs_{unstimulated\,cells}}}{{Abs_{LDH\,positive\,control}}}x100$$

### Cytokine release assay

The concentrations of IL-1β and IL-18 were measured in cell culture supernatants using the Mesoscale MSD U-Plex platform following the manufacturer’s instructions. Plates were read using an MSD 1300 microplate reader at the University of Liverpool Shared Research Facilities.

### Quantification of inflammasome assembly

So-called ASC specks resemble oligomerized inflammasomes. Specks released into cell culture supernatants through pyroptosis were quantified following an adapted version of the protocol from Rowczenio et al. [16] on the Guava flow cytometer. Data were analysed using Kaluza software (Beckman Coulter, Life Sciences). Within each experiment, ASC speck counts were normalised to unstimulated controls; speck counts are displayed as a percent change compared to unstimulated controls over the total amount of events (10.000).

$${\it{Percent}}\,{\it{change}} = \frac{{{\it{Counted}}\,1\,\mu {\it{m}}\,{\it{particles}}({\it{sample}} - {\it{control}}){\it{x}}\,100}}{{10.000}}$$

### Data analysis

Data were graphed and analysed using GraphPad Prism 9 (San Diego, California, USA). Statistical significance was tested using Kruskal–Wallis, Wilcoxon, or Mann–Whitney tests as indicated. Differences with a *p* ≤ 0.05 were considered statistically significant. All experiments were independently repeated at least four times and are represented as boxplots showing median and interquartile range.

## Results

### NaCl induces cell death, IL-1β and IL-18 release in primary human monocyte-derived macrophages

**Fig. 1:**
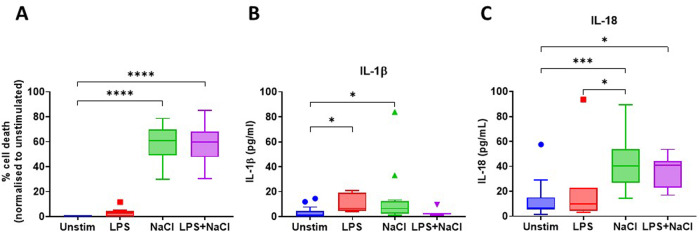
NaCl alone or with LPS induces inflammatory cell death, IL-1β and IL-18 release in primary human monocyte-derived macrophages (MDM). **A** Inflammatory cell death evaluated through LDH assay after 8 h of stimulation; IL-1β (**B**) and IL-18 (**C**) release measured through MSD assays. LPS lipopolysaccharide, Unstim Unstimulated cells. *N* > 9; Kruskal–Wallis test. *: *p* < 0.05; ***: *p* < 0.001; ****: *p* < 0.0001.

To evaluate the effect of hypertonic saline on primary human monocyte-derived macrophages (MDMs), cells were stimulated for 8 hours with 500 mM NaCl (approximately 3% NaCl) in the absence or presence of LPS to mimic bacterial colonisation. After stimulation, cell culture supernatants were collected for LDH and MSD assays. Hypertonic saline alone (60.9% versus untreated, *p* < 0.0001) or in combination with LPS (59.7% versus untreated, *p* < 0.0001) induced inflammatory cell death, IL-1β release (NaCl: 1.18 vs 6.58 pg/mL, *p* = 0.02; NaCl + LPS: 1.18 vs 2.34 pg/mL, *p* > 0.99) and IL-18 release (NaCl: 6.85 vs 40.42 pg/mL, *p* < 0.001; NaCl + LPS: 6.85 vs 40.72 pg/mL, *p* = 0.02) (Fig. 1).

### Anakinra and MCC950 do not alter NaCl-induced inflammation in primary human monocyte-derived macrophages

**Fig. 2:**
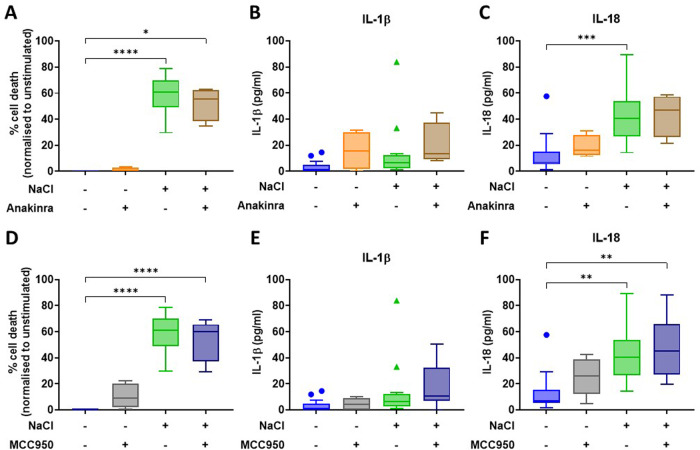
Anakinra and MCC950 do not alter NaCl-induced inflammation in primary human MDM. Supernatants were analysed after 8 h of stimulation. **A**, **D** Inflammatory cell death evaluated through LDH assays; **B**, **E** IL-1β concentrations measured through MSD assays; **C**, **F** IL-18 concentrations measured through MSD assays. *N* ≥ 4; Kruskal–Wallis. *: p < 0.05; *: *p* < 0.01; ***: *p* < 0.001; ****: *p* < 0.0001.

To decipher underlying molecular mechanisms, the recombinant IL-1 receptor antagonist anakinra or the small molecule NLRP3 inhibitor MCC950 were added to cell culture assays (Fig. 2). Neither NaCl-induced cell death nor pro-inflammatory cytokine release were altered by these inhibitors (all *p* > 0.1).

### NaCl induces the release of ASC specks in primary human monocyte-derived macrophages

**Fig. 3:**
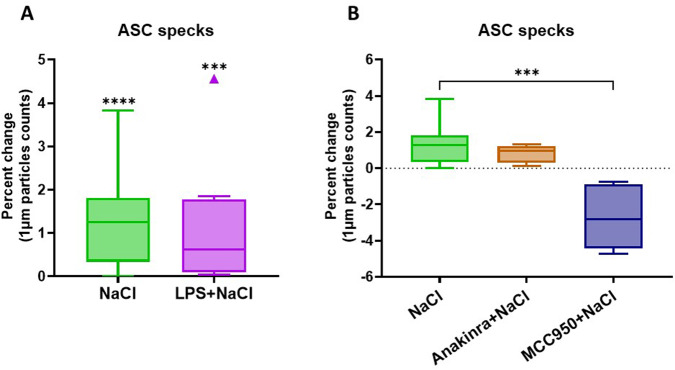
The stimulation with NaCl induces the release of ASC specks in primary human MDM. **A** ASC specks in cell culture supernatants following 8 h of stimulation with LPS, NaCl, or NaCl and LPS, were quantified via flow cytometry and normalised to the unstimulated controls; **B** MCC950, but not Anakinra, reverts NaCl effect, reducing the amount of ASC specks, in a biologically meaningful manner; data were normalised to the relative unstimulated control. *N* > 4; Kruskal–Wallis test. ***: *p* < 0.001; ****: *p* < 0.0001.

To further study the effect of hypertonic NaCl on primary MDMs, we quantified the release of assembled inflammasomes (ASC specks) into cell culture supernatants using flow cytometry. After 8 hours of stimulation, NaCl alone (*p* < 0.001) or in combination with LPS induced ASC speck release (*p* < 0.001) (Fig. 3A). As the treatment with NaCl alone resulted in most pronounced effects on cell death and cytokine release (Fig. 1), we focused our attention to this condition when investigating effect of anakinra and MCC950. Notably, while culture with anakinra did not alter ASC speck release (*p* > 0.9), treatment with MCC950 reduced it by approximately 4% (*p* < 0.001) (Fig. 3B).

### Hypertonic NaCl induces cell death and IL-1β release in THP-1 derived macrophages

**Fig. 4:**
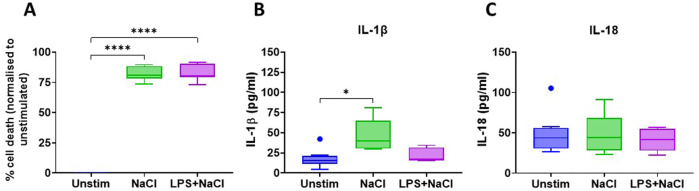
Hypertonic saline induces inflammatory cell death and IL-1β release in THP-1 derived macrophages. Inflammatory cell death, IL-1β and IL-18 release were evaluated after 8 h of stimulation. **A** Cell death measured via LDH assay; data were normalised to the unstimulated controls; **B**, **C** IL-1β and IL-18 concentrations respectively measured through MSD assays; *N* ≥ 7; statistical analysis performed with Mann–Whitney test. *: *p* < 0.05; ****: *p* < 0.0001.

To test whether THP-1 derived macrophage-like cells could be used as an easily accessible model system, we repeated the experiments in these cells. Similar to primary human MDM, NaCl-induced inflammatory cell death in these cells (80.25%, *p* < 0.001) and induced the release of activated/mature IL-1β (15.65 vs 39.84 pg/mL, *p* = 0.01); in contrast, IL-18 release was not increased after treatment with NaCl (43.71 vs 44.21 pg/mL, *p* > 0.9) (Fig. 4).

### Anakinra and MCC950 do not alter NaCl-induced inflammation in THP-1-derived macrophages

**Fig. 5:**
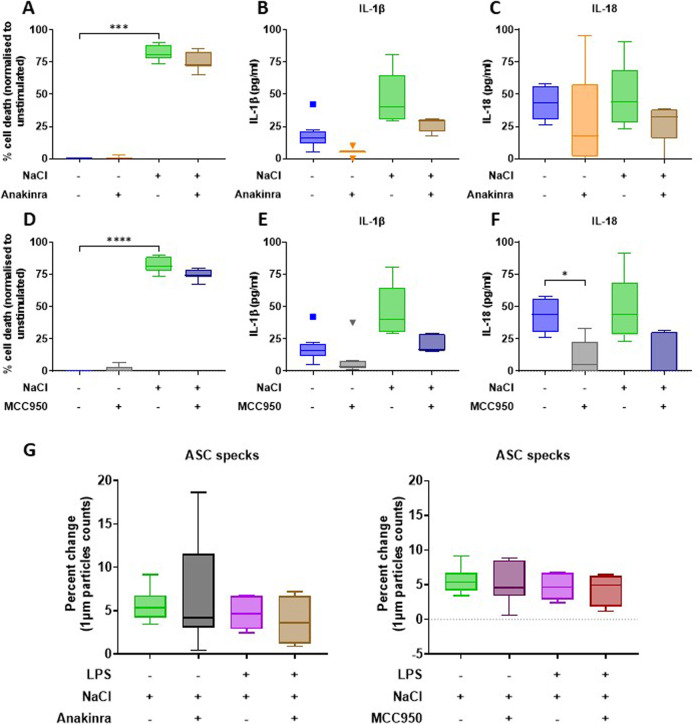
Anakinra and MCC950 do not alter NaCl-induced inflammation in THP-1 derived macrophages. Supernatants were analysed after 8 h of stimulation. **A**, **D** Inflammatory cell death evaluated through LDH assays; **B**, **E** IL-1β concentration measured through MSD assays; **C**, **F** IL-18 concentrations measured through MSD assays; **G** ASC specks in cell supernatants following 8 h of stimulation with NaCl, or NaCl and LPS, were quantified via flow cytometry and normalised to unstimulated controls. Data were normalised to the relative unstimulated control. *N* ≥ 4; Kruskal–Wallis. ***: *p* < 0.001; ****: *p* < 0.0001.

Next, we quantified cell death, and IL-1β, IL-18 and ASC specks release from THP-1-derived macrophage-like cells into cell culture supernatants in the absence or presence of anakinra or MCC950 (Fig. 5). Notably, none of the inhibitors reversed NaCl effects, again suggesting that this phenomenon might not be NLRP3 dependent (all *p* > 0.1).

### Knock-down of NLRP1 inhibits NaCl-induced release of IL-1β and ASC specks

**Fig. 6:**
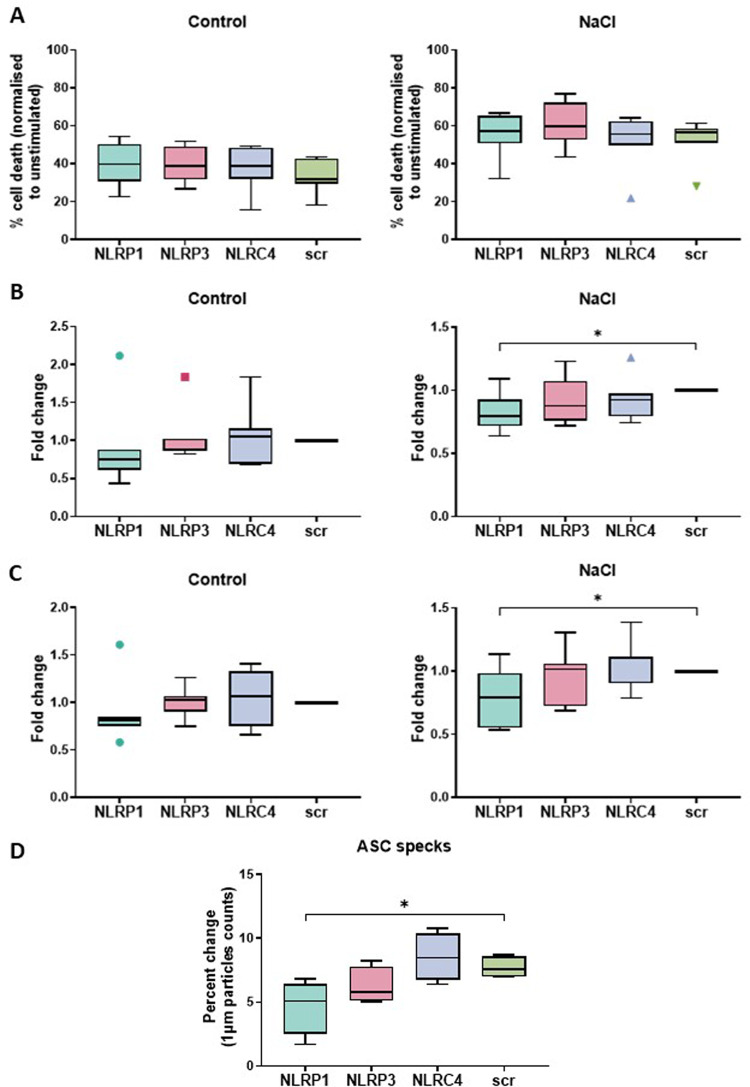
*NLRP1* knock-down inhibits NaCl-induced release of IL-1β, IL-18, and ASC specks. **A** Inflammatory cell death evaluated through LDH assays; **B**, **C** IL-1β and IL-18 measured through MSD assays; **D** Extracellular ASC specks quantified via flow cytometry and normalised to the unstimulated controls. Statistical significance was calculated using the Mann–Whitney test; *N* ≥ 4; *: *p* ≤ 0.05.

To determine which inflammasome was activated by hypertonic saline, we transfected THP-1-derived macrophages with siRNAs targeting *NLRP1*, *NLRP3*, or *NLRC4*, and repeated LDH, cytokine and ASC speck release assays (Fig. 6). While knock-down of *NLRP3* or *NLRC4* had no effects, *NLRP1* down-regulation reduced IL-1β, IL-18, and ASC specks release into cell culture supernatants (all *p* < 0.05) (Fig. 6B–D), suggesting NLRP1 inflammasome involvement in the response to NaCl.

## Discussion

Inhalation of hypertonic saline solution (3–7%) can increase the clearance of airways and is, therefore, routinely used in patients with CF and bronchiolitis [1]. However, altered ion and/or salt concentrations can trigger inflammasome activation, inflammation and tissue damage [10–14]. Furthermore, increased dietary salt intake may be involved in the onset and exacerbation of autoimmune/inflammatory diseases, especially through its effects on macrophages [17, 18]. This study shows that NaCl triggers inflammasome assembly in primary human monocyte-derived macrophages (MDMs), causing inflammatory cell death, IL-1β, IL-18 and ASC speck release. Notably, ASC specks release can contribute to the propagation of inflammation as they can be passed on to other phagocytic cells, and recruit and activate procaspase-1 [19].

This study showed that knock-down of *NLRP1* reduced release of IL-1β, IL-18 and ASC specks into cell culture supernatants, suggesting that the NLRP1 inflammasome is involved in the stress response to hypertonic NaCl (3% solution). Moreover, no substantial effects were detected in response to culture of cells with the recombinant IL-1 receptor antagonist anakinra or the small molecule NLRP3 inhibitor MCC950. The 4% reduction of ASC release after treatment with MCC950 may, though statistically significant, likely not be biologically meaningful. This would suggest that NLRP3 inflammasome is not centrally involved in the inflammatory response to hypertonic NaCl under the here investigated conditions.

Although this study has provided insights into the molecular mechanisms underlying the pro-inflammatory effects mediated by hypertonic NaCl in human macrophages, it has several limitations. While exhibiting similar effects of hypertonic NaCl on inflammatory cell death, IL-1β and ASC speck release, differences between primary MDMs and THP-1-derived macrophages were seen in relation to IL-18 and effects of MCC950 on ASC speck release. Other studies have shown differential effects of stimuli by MDMs and THP-1-derived macrophages, especially with regards to cytokines secretion [20, 21]. We decided to use THP-1-derived macrophages as a model system for knock-down experiments because inflammatory cell death, IL-1β and ASC speck release in response to hypertonic NaCl were somewhat comparable.

Lastly, this study focused on macrophages and did, at this stage, not include additional cell types. Future studies will investigate the effect of NaCl on airway epithelia and additional immune cells, also following infection with respiratory viruses. In conclusion, NaCl induces inflammatory cell death, inflammatory cytokines and ASC speck release in primary MDMs and THP-1-derived macrophages through the activation of NLRP1 inflammasomes. Therapeutic inhibition of inflammasome assembly may therefore improve disease outcomes in conditions where inhalation of hypertonic saline is used for mucus mobilization.

## Data Availability

The data that support the findings of this study are available from the corresponding author upon reasonable request.
